# Molecular, proteomic and immunological parameters of allergens provide inclusion criteria for new candidates within established grass and tree homologous groups

**DOI:** 10.1186/s40413-015-0069-9

**Published:** 2015-07-16

**Authors:** Matthew D Heath, Joe Collis, Toby Batten, James W Hutchings, Nicola Swan, Murray A Skinner

**Affiliations:** Allergy Therapeutics Ltd., Dominion Way, Worthing, BN14 8SA UK

**Keywords:** Homology, Allergens, Grass, Tree, Structure, Cross-reactivity

## Abstract

**Background:**

Our knowledge of allergen structure and function continues to rise and new scientific data on the homology and cross-reactivity of allergen sources should be considered to extend the work of Lorenz *et al.*, 2009 (Int Arch Allergy Immunol. 148(1):1–1, 2009) and the concept of homologous groups. In addition to this, sophisticated techniques such as mass spectrometry (MS) are increasingly utilised to better characterise the complex mix and nature of allergen extracts.

**Methods:**

Homology models were used of Fag s 1 (Beech) and Cyn d 1 (Bermuda grass) and compared with template crystal structures of Bet v 1 and Phl p 1 from the ‘exemplar’ species of Birch and Timothy grass, respectively. ELISA experiments were performed to assess cross-reactivity of Beech (tree) and Bermuda (grass) extracts to rabbit sera raised to either “3-Tree” (Birch, Alder and Hazel) extract or “Grass” (12-grass mix extract), respectively. The comparability of biochemical stability of different allergen sources was assessed through statistical methods for a range of tree and grass species.

**Results:**

Allergen cross-reactivity and/or structural homology have been described providing justification for inclusion of Beech within the Birch homologous tree group. Data from Bermuda grass (Cyn d 1) provides further justification for the inclusion of this species into the homologous group of the sweet grasses. However, further characterisation of relevant allergens from Bermuda grass and, in particular, comparison of cross-reactive patterns between subjects specifically in areas with high abundance of both *Pooideae* and *Chloridoideae* is sought.

**Conclusion:**

MS allows the possibility to identify individual proteins or allergens from complex mixes by mass and/or sequence, and this has been extensively applied to the allergen field. New data on the homology, cross-reactivity and biological parameters of allergen sources have been considered to extend the work of Lorenz *et al.*, 2009 in the context of tree and grass species. The concept of homologous groups is certainly dynamic allowing the flexibility and potential in streamlining quality parameters, such as stability profiles, due to extrapolation of exemplar data to a wider range of allergens.

**Electronic supplementary material:**

The online version of this article (doi:10.1186/s40413-015-0069-9) contains supplementary material, which is available to authorized users.

## Background

### Allergen characterisation and the concept of homologous groups

The concept of ‘homologous groups' for allergen products was established (among pollens and mites) each of which identifies a representative allergen source based on the wealth of empirical data relating to structural homology and cross-reactivity [1]. In the “*Guideline on Allergen Products: Production and Quality Issues”*, the EMA adopted this concept of grouping allergen extracts according to defined and justified scientific criteria [2]. Allergen extracts from different species, genera or families may be included into homologous groups, providing all of the following criteria are fulfilled:

- Comparable biological and physicochemical properties of the source material
- Allergen structural homology/cross-reactivity
- Identical formulation of the finished product
- Identical production process of the allergen extract and of the finished product

Our knowledge of allergen structure and function continues to rise and new scientific knowledge on the homology and cross-reactivity of allergen sources should be considered to extend the work of Lorenz *et al.*, 2009 [1]. In addition to this, sophisticated techniques such as mass spectrometry (MS) have been recently utilised to better characterise the complex mix and nature of allergen extracts [3]. While further development of immunoassays and their application to larger data sets will reveal additional insights into allergen cross-reactivity profiles, MS allows the possibility to identify individual proteins or allergens from complex mixes by mass and/or sequence, and this has been extensively applied to the allergen field [3].

In each homologous group allergens can be selected as a representative, or ‘exemplar’ for the group, allowing extrapolation of data to other group members [1,2]. Therefore, this concept could be particularly useful in streamlining quality parameters, such as stability profiles, due to extrapolation of exemplar data to a wider range of allergens. At the time of this proposal for homologous grouping, for several allergen species insufficient data were available to justify inclusion into the established homologous groups and these species were classified as ‘non-grouped’. Lorenz and colleagues, 2009 state: *“The concept of homologous groups is dynamic and groups could be complemented by additional species depending on the availability of new information on identified allergens, homologous allergen families and cross-reactivities.”*[1].

### Tree species

Three groups of homologous tree species were outlined in Lorenz *et al.*, 2009 (Birch, Oleaceae, Cupressaceae). A number of tree pollen species could not be assigned to any of the three established groups due to the absence of scientific information and, therefore, lack of justification: Maple, Poplar, Willow, Elm, Locust, Linden, Plane and Beech. Of these, only Beech and Plane species have undergone further characterisation and sequencing with the major relevant allergen in Beech (Fag s 1) identified and several allergens from Plane pollen characterised (i.e. Pla a 1, Pla a 2, Pla a 3) [4]. However, no crystal structure currently exists for the aforementioned allergens. Cross-reactivity studies of Plane with other tree pollen species did not sufficiently demonstrate an immunological relationship of the allergens that would allow a group formation. While in relation to Beech, Lorenz *et al.*, 2009 state: *“…skin tests indicated that beech, belonging to the order Fagales, is a possible candidate for the ‘birch group’, but at the present time the information is not substantial enough to justify this classification…”*[1]*.* However, it is not elaborated to what extent scientific information is lacking.

Beech pollen has a wide distribution notably in Central and Southern Europe, North America, Asia and Africa [5]. Current evidence suggests that allergy to Beech pollen is mainly due to cross-reactivity with Birch pollen allergens [6]. Five different tree species (Birch, Alder, Hazel, Oak, Hornbeam) of the order *Fagales* are currently represented in the Birch group. The best characterised species within this group, Birch, represents the exemplar species [1]. The best-studied allergenic representative of this family is the group 1 allergen Bet v 1, with the majority of patients sensitized towards this allergen [7].

The striking similarities in structural homology and cross-reactivity of the named species representing this group illustrate why they fulfil the criteria for the formation of the proposed Birch group [1]. The defined criteria outlined by the EMA guidance allows for the possible addition of Beech pollen - providing current scientific information support this in a reasoned manner, of which is the primary objective of this report.

### Grass species

In various sweet grasses of the Poaceae (Gramineae) family each species contains varieties of allergens and variants of each (isoallergens). The prevalence and distribution of grasses will undoubtedly impact patterns of sensitization and cross-reactivity. It has also been suggested on a number of occasions that a broad-spectrum product comprising 13 different grass species better mimics natural exposure conditions for a patient and is proven to be suitable for the treatment of allergic rhinitis [8].

The best characterised species within the grass homologous group, Timothy grass, represents an exemplar species [1]. The major relevant allergens in this instance (e.g. Phl p 1) are well characterised enabling structural homology to be appropriately assessed [9-13]. The striking similarities in structural homology and cross-reactivity of the named species representing this group illustrate why they fulfil the criteria for the formation of the proposed homologous group. However, Bullimore *et al.*, 2012 questioned whether the current strategy for dealing with data extrapolation from one species to another, in terms of homologous groups, is appropriate. In this study, the authors suggested that any member of the group could act as the exemplar species since none of the grass species examined displayed identical biological profiles, while providing a justification for the inclusion of Crested Dogstail [8]. Ongoing work in the field seeks to further characterise the cross-reactivity through analysing large datasets of IgE cross-reactivity towards different species [14]. The grass species reviewed in this study is Bermuda grass and its major relevant allergen – Cyn d 1.

Lorenz and colleagues, 2009 argue that cross-reactivity of Cyn d 1 with other group 1 grass allergens is contradictory, citing a number of studies over the past decade. Of the seven characterised Bermuda grass allergens, only two appear to be cross-reactive (Cyn d 1 and Cyn d 7) but present contradictory results [1,15-17]. However, cross-reactivity studies have since been explored including a comparison of allergen-specific IgE binding from different grass species by Johansen *et al.*, 2009, using patient sera from thousands of subjects from North America and Europe. Correlation was observed between the IgE response to Bermuda grass and Timothy grass in sera from grass pollen allergic patients; part of this is explained by the existence of shared epitopes for allergens of the Pooideae and Chloridoideae subfamilies [14].

The aim of this report is to provide an essential framework process and enable a scientifically credible route to assess inclusion criteria for new candidates within established grass and tree homologous groups, in line with EMA guidance. In addition to providing a useful and original example of how one might achieve this through the review in the current status of allergen characterisation and utilisation of molecular and computational approaches currently applied in the field, with focus on two major relevant allergens from Beech (Fag s 1) and Bermuda grass (Cyn d 1).

## Methods

### Proteomics

Aqueous pollen extracts of Birch, Alder, Hazel, Oak, Beech, Timothy grass and Bermuda grass were each treated with dithiothreitol and heat-denatured at 70°C for 30 minutes in sample buffer containing sodium dodecyl sulfate. Samples were then applied to a 10%–20% Tris–HCl Criterion gel (Bio-Rad, Hemel Hempstead, UK) and electrophoresed according to the manufacturer’s protocol. Coomassie blue-stained protein bands corresponding to major allergens Bet v 1, Cor a 1, Aln g 1, Que a 1, Fag s 1, Phl p 1 and Cyn d 1 were excised from the gel and provided to the Central Proteomics Facility at the University of Oxford for purification, tryptic digest and analysis by tandem mass spectrometry. Using the Mascot server [18], allergens were identified against NCBI and SwissProt databases [19,20].

### Protein homology modelling

The primary sequence for Beech major allergen, Fag s 1, was retrieved from the UniProt database using the accession code B7TWE6. A homology model of Fag s 1 was generated using SWISS-MODEL with the crystal structure of Bet v 1 (PDB ID: 1BV1) as a template structure [21]. The homology model of Cyn d 1 was retrieved from SDAP (model 101). Structures were presented and visualised using Chimera v1.10 [22]. PROCHECK analysis gave an indication of good stereochemical quality of the structure and overall residue-by-residue geometry [23].

### Statistical analysis of biochemical properties

Comparability of protein content stability from different allergen extracts was assessed through statistical methods for a range of allergens. 36 month data from extracts of source material were required for the exemplar allergen. To allow direct comparison of different allergens with different target concentrations the proportional change from baseline in the parameter result was used as the response variable. The proportional change from baseline was analysed in SAS v9.3 using an analysis of covariance (ANCOVA) with allergen as a fixed effect, batch as a random effect and time in days from baseline testing as a covariate and the interaction effect between allergen and time in days from baseline testing. Separate models were used for each parameter. The significance of the interaction effect at the 25% significance level was used to compare the stability profiles for each allergen. The significance level was selected in accordance to ICH Q1E *“Evaluation for Stability data”* [24].

### Determination of allergen cross-reactivity

ELISA experiments were performed to assess cross-reactivity of Beech (tree) and Bermuda (grass) allergen extracts to rabbit sera raised to either “3-Tree” (Birch, Alder and Hazel) extract or “Grass” (12 grass mixes extract), respectively (note: species are presented in Table 3, with the addition of Crested Dogstail). Allergen extract preparations were diluted with water to equivalent protein content and applied to a microtitre plate. For the tree experiments, sera raised to Ragweed, and a Ragweed sample, were used as a control. For the Grass experiments, sera raised to Olive and an Olive sample was used as a control. Plates were washed with 0.1% v/v Tween-20 in DPBS, blocked with 2% glycine in DPBS and resolved with an anti-rabbit IgG-alkaline phosphatase secondary antibody and phosphatase substrate. Plates were incubated at 37°C for 30 minutes between each step, and read at 405 nm. The absorbance of each of the samples was then compared to the exemplar.

## Results

### A molecular approach to allergen characterisation

**Figure 1:**
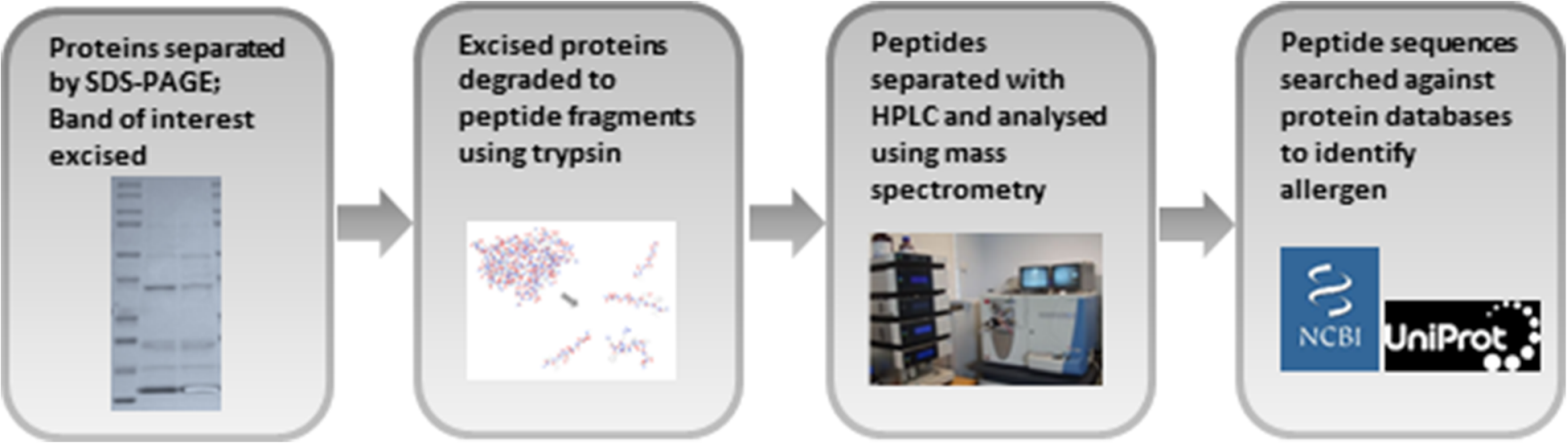
Allergen sample preparation and analysis.

MS has been extensively applied to allergen extracts in the field, in order to verify relevant allergens from these complex mixtures. This can be applied to different groups ranging from plants through to food groups and animal hairs. The samples were prepared and then analysed by mass spectrometry according to Figure 1.

Table 1 presents the sequence coverage scores of major relevant allergens from a selection of monocotyledons and dicotyledons pollen extracts.

**Table 1:** **Allergen verification using MS of a selection of mono- and dicotyledon allergen extracts**

	**Allergen (kDa)**	**No. tryptic peptides**	**Sequence coverage (%)**
**Monocotyledons**			
Timothy Grass	Phl p 1 (32 kDa)	13	58 %
Bermuda Grass	Cyn d 1 (32 kDa)	7	32 %
**Dicotyledons**			
Birch	Bet v 1 (16 kDa)	15	91 %
Alder	Aln a 1 (16 kDa)	11	71 %
Hazel	Cor a 1 (16 kDa)	9	59%
Oak	Que a 1 (16 kDa)	10	67 %
Beech	Fag s 1 (16 kDa)	11	57 %

Note: A summary of the current status in quantification of major relevant allergens that have been previously performed are reviewed in Chapman *et al.*, 2012 and Batard *et al.*, 2014 ([3], [30], respectively).

### Further insight into existing homologous groups in allergen products

A number of grouped and non-grouped tree and grass pollen extracts were assessed against the criteria outlined in the EMA *Guideline on Allergen Products* [2]. All products implicated in this study are formulated using an identical process to give an identical finished extract, bar allergen type. Allergen cross-reactivity and structural homology were investigated by immunoassays and computational methods, respectively. In addition to this, the comparability of biochemical properties was assessed through analysing protein content stability profiles of different allergen species using statistical methods for a range of allergens.

#### The ‘Birch group’

The structure and allergenic potential of the clinically most important *Fagales* pollen allergen from Beech (Fag s 1) has since been further characterised herein and in the current literature [25]. This, in addition to their structural homology, cross-reactivity and biochemical profiles between the Birch group and Beech are presented.

The sequence identity of Fag s 1 (65%) is somewhat lower than the group 1 allergens in Alder, Hazel and Hornbeam compared with Birch Bet v 1 (79-83%). However, Fag s 1 exhibits a higher sequence identity to Bet v 1 than Que a 1 (Oak) (58%), which is already included in the Birch homologous group (Table 2).

**Table 2:** **The ‘Birch homologous group’ and their sequence identities to Bet v 1**

**MW kDa**	**Major relevant allergen**	***Betula verrucosa*** **(Birch)**	***Alnus glutinosa*** **(Alder)**	***Corylus avellana*** **(Hazel)**	***Quercus alba*** **(Oak)**	***Carpinus betulus*** **(Hornbeam)**
16	Bet v 1	Bet v 1	Aln g 1	Cor a 1	Que a 1	Car b 1
		100%*	83%	81%	58%	79%

*sequence identity.

Note: Fag s 1 (Beech) has a sequence identity to Bet v 1 (birch) of 69%.

(i) Structural homology

Proteomic analysis of in-house commercial Beech extracts has been analysed and interpreted. The major relevant allergen has been identified as Fag s 1 at 16 kDa with 57% coverage of the primary sequence confirmed.

**Figure 2:**
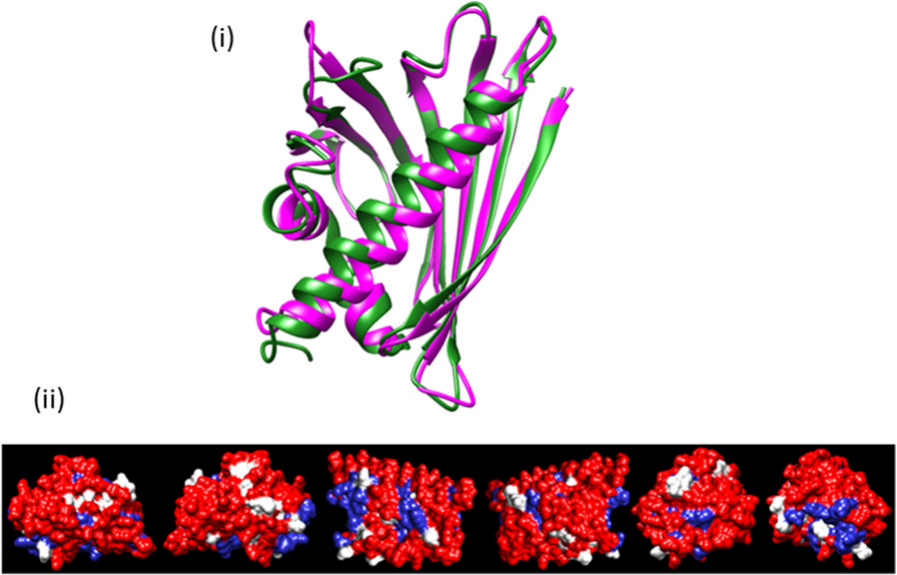
Structural homology of Fag s 1. **(i)** Superimposed Ribbon drawing of the Fag s 1 (green) homology model superimposed onto the template crystal structure of Bet v 1 major allergen (magenta) **(ii)** Space filling model of Fag s 1; conserved amino acid residues are coloured in red, different residues in white, homologous substitutions in blue (69% identity/80% similarity).

The homology model of Fag s 1 is identical in its overall fold/topology with the known crystal structure of Bet v 1; exhibiting a characteristic 6 anti-parallel β-sheet topology with 3 alpha-helical segments (Figure 2).

The global fold is dominated by 4 beta-strands and 2 of the helices form a C-terminal amphipathic helical motif, representing true structural homology. The Root Mean Squared Deviation (RMSD) of the modelled Fag s 1 structure is 1.48 Å. In addition to this, the sequence alignment (not shown) reveals no notable insertions/deletions.

(ii) Cross-reactivity

**Figure 3:**
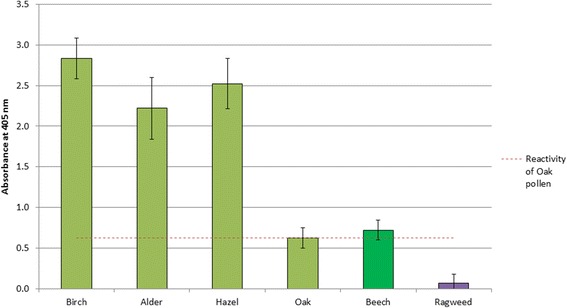
Cross-reactivity of grouped and ungrouped (Beech) trees against 3-Tree (Birch, Alder, Hazel) positive rabbit sera. Error bars represent the standard deviation from 4 replicates.

An ELISA experiment was performed to assess cross-reactivity of each of the currently grouped tree extracts (Birch, Alder and Hazel) compared to Beech using rabbit sera raised to a 3-Tree (Birch, Alder and Hazel) extract. The absorbance of each of the samples was then compared to that for the exemplar (Birch), presented in Figure 3.

The similarity in cross-reactivity of Beech to Birch, Alder and Hazel is greater than that of Oak, which is a Birch-grouped tree species. All tree species showed negligible reactivity to Ragweed (negative control) sera (additional file 1).

(iii) Statistical analysis of biochemical properties

The comparability of protein content stability profiles from different sources of extract was assessed through statistical methods for a range of tree extracts. To determine the similarity of stability profiles, stability data collected over a 36 month period for tree allergen extracts were pooled for analysis. The parameters considered for the analysis was protein content, to determine biochemical similarity in this context.

For the comparison of allergens within the proposed Birch group, Alder and Hazel allergen extracts supplemented the available Birch stability data. The protein content stability profiles demonstrate comparability between the different extracts tested.

**Figure 4:**
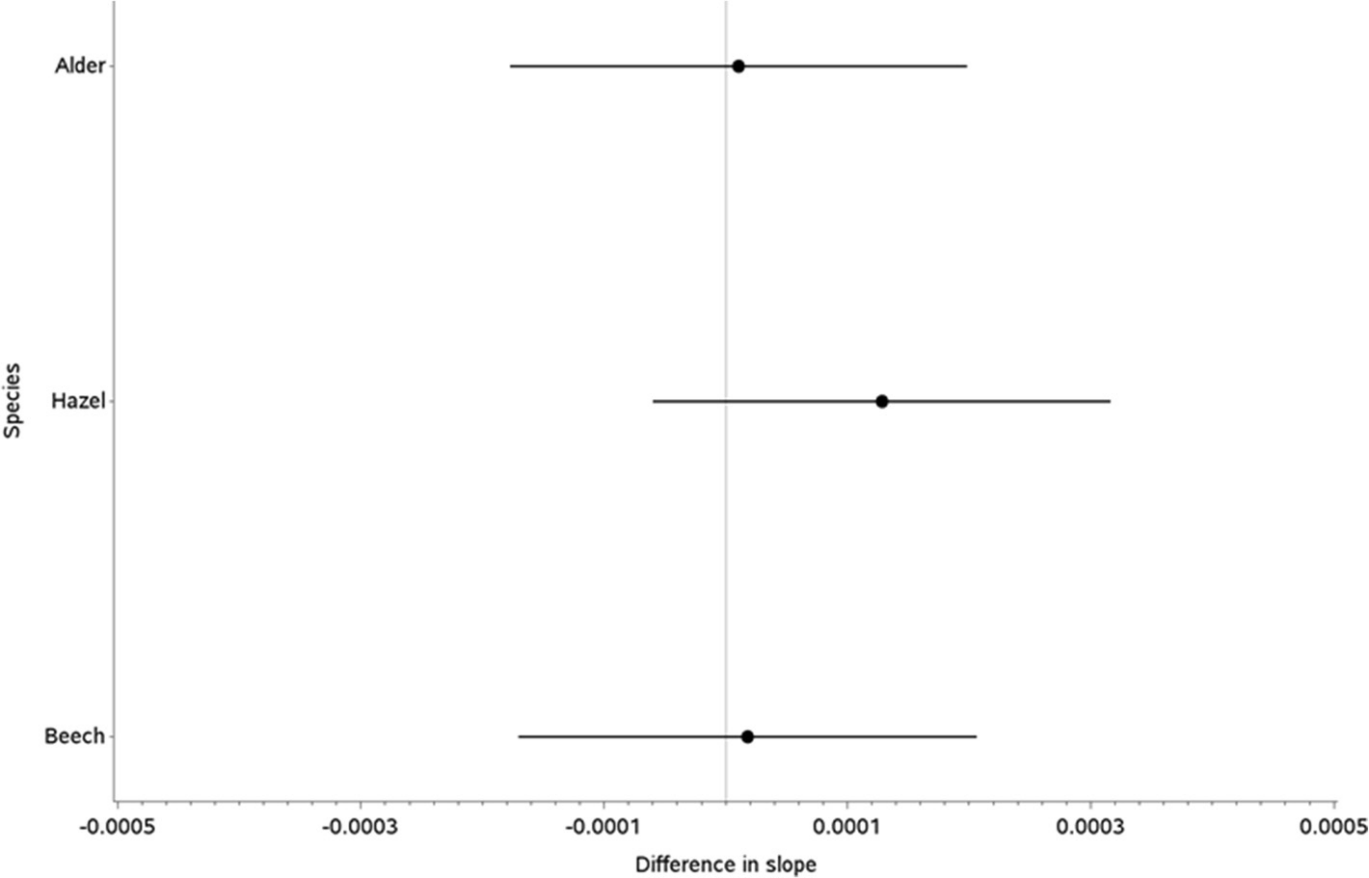
Species and time interaction effect coefficients and 95% confidence intervals for protein content (using Birch as reference).

The p-value for the allergen and time interaction effect was 0.6571, thus meeting the grouping criteria. The different interaction effects and 95% confidence intervals are displayed graphically in Figure 4. There are no significant differences in the protein content stability profile between each pollen species. Beech appears to have a very similar trend to both Birch and Alder. Furthermore the statistical analysis does not show any significant differences in the intercepts for each allergen (p = 0.4445).

### The group of Sweet Grasses

The group of sweet grasses of the Poaceae (Gramineae) family, the subfamily Pooideae, is characterised by Timothy grass (Phl p 1) as the exemplar species. The established homologous group for grass allergens is presented in Table 3. Further characterisation of Bermuda grass is presented herein.

**Table 3:** **Current homologous group for Grass allergens (exemplar species in bold)**

**Homologous group**	**Established allergen species (Lorenz** ***et al.*** **, 2009)**
Grass/Cereal pollen	*Anthoxanthum odoratum* Sweet vernal grass
	*Avena sativa* Oat
	***Dactylis glomerata*** **Orchard grass/cocksfoot**
	*Festuca* spp. Meadow fescue
	*Holcus lanatus* Velvet grass/Yorkshire fog
	*Hordeum vulgare* Barley
	*Lolium perenne* Perennial ryegrass
	***Phleum pratense*** **Timothy grass**
	***Poa pratensis*** **Kentucky bluegrass**
	*Secale cereale* Cultivated rye
	*Triticum aestivum* Cultivated wheat

(i) Structural homology

The structure and allergenic potential of the clinically relevant allergen from Bermuda grass (Cyn d 1) has been further characterised in the literature [16]. The differences in sequence identities of the two isoforms of Cyn d 1 were highlighted on the crystal structure of the exemplar group 1 allergen from Timothy grass - Phl p 1 [16]. The sequence identity of Cyn d 1a and Cyn d 1b is moderate-high (71 and 68% respectively).

**Figure 5:**
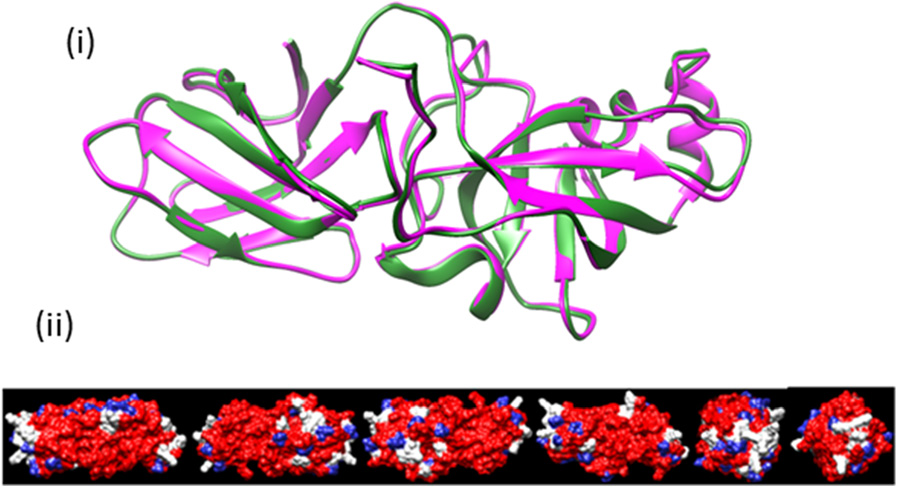
Structural homology of Cyn d 1. **(i)** Superimposed Ribbon drawing of the Cyn d 1 (green) homology model superimposed onto the template crystal structure of Phl p 1 major allergen (magenta) **(ii)** Space filling model of Cyn d 1, conserved amino acid residues are coloured in red, different residues in white, homologous substitutions in blue (68% identity/76% homology).

The ribbon drawing in Figure 5 presents a homology model of Cyn d 1 (SDAP model 101) superimposed onto Phl p 1 (PDB: 1 N10) from the exemplar species Timothy grass.

The homology model of Cyn d 1 reveals an identical overall fold/topology, with an almost exclusive β-sheet secondary topography and identical two-domain organisation. The RMSD of the modelled Cyn d 1 structure was 0.5 Å. Note: the template structure of Phl p 1 presents no structure residues between amino acids 29–38, and likewise in the modelled structure.

(ii) Cross-reactivity

**Figure 6:**
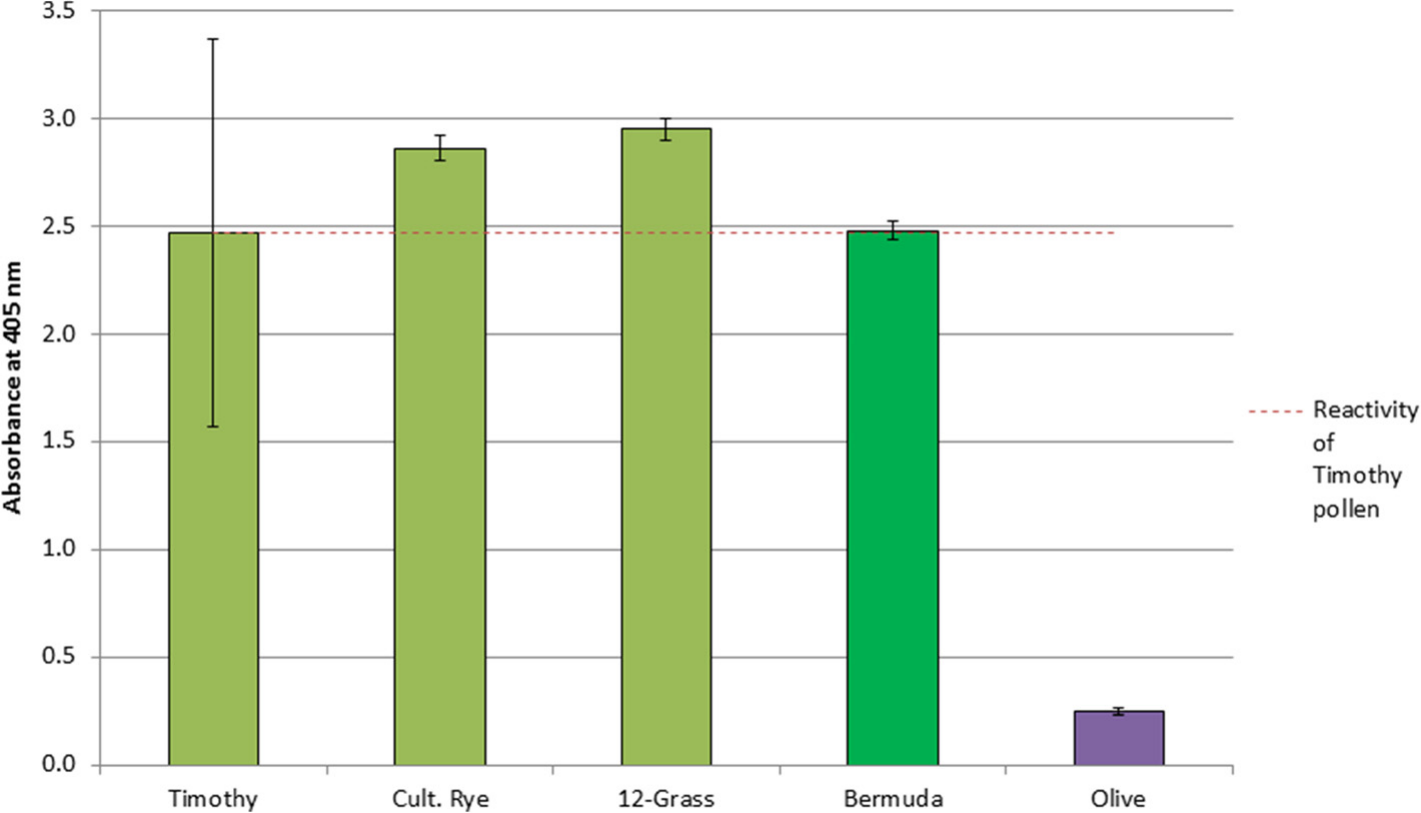
Cross-reactivity of grouped and non-grouped (Bermuda) grass species against 12-grass positive rabbit sera. Error bars represent the standard deviation from 4 replicates.

An ELISA was performed to assess cross-reactivity of each of the Grass extracts to rabbit sera positive to “Grass” (12-Grass mix) extract, where the 12-grass mix includes 11 of the considered homologous group grass species plus Crested Dogstail grass which has been identified as a candidate for grouping by Bullimore *et al*., 2012 [8] (Figure 6).

All grasses showed negligible reactivity to Olive (negative control) sera (additional file 2). The similarity in cross-reactivity of Bermuda grass is comparable to Timothy grass which represents the exemplar species in this group.

(iii) Statistical analysis of biochemical properties

The comparability of protein content stability profiles from different sources of extract was assessed through statistical methods for a range of grass extracts. To determine the similarity of stability profiles, stability data collected over a 36 month period for grass allergen extracts were pooled for analysis. The parameters considered for the analysis was protein content, to determine biochemical similarity.

**Figure 7:**
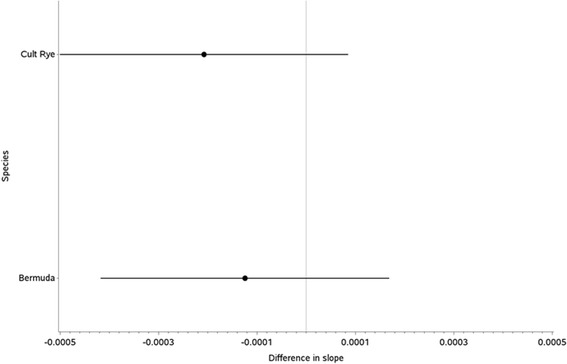
Allergen and time interaction effect coefficients and 95% confidence intervals for protein content (using 12-grass as reference).

For the comparison of allergens within the proposed group, the available protein content stability data was available for Cultivated Rye, Bermuda grass and 12-grass extracts. The p-value for the allergen and time interaction effect was 0.2602, thus meeting the grouping criteria. Figure 7 shows there are no significant differences in the protein content stability profile between Cultivated Rye and Bermuda grass.

## Discussion

### Allergen verification

Data generated from allergen verification programmes is currently used to facilitate the identification and characterisation of relevant allergens. This is now commonly achieved through applying proteomics mass spectrometry to complex allergenic mixtures. It is clear that MS is a powerful tool for identification of allergens which is consistently demonstrated using relevant grass and tree sources presented herein.

Knowledge of protein structure is key to understanding functional and evolutionary relationships between allergens. Common ancestry (i.e. homology) can be more accurately inferred through their structural alignments and subsequent domain annotations. Grouping allergens into families (using their domain assignments) allows us to more accurately assess patterns of species distribution and relationships. Recent efforts to include structural information on allergens in predicting cross-reactivity have involved classification into Pfam classes [26,27]. Results presented herein and in the relevant literature reveal that most allergens from a wide variety of different sources are found in a relatively limited number of protein families [27]. Families important in pollen include Bet v 1 homologues (PR-10), Profilins (both of which are known to be responsible for pollen-associated food allergies), B-expansins (Phl p 1) and Trypsin inhibitor (Ole e 1). Whereas Lipocalins, cytoskeletal proteins, transport proteins, polysaccharides, storage and transport proteins define the general biochemical function of known allergens from other sources such as epithelials, nuts/grains.

### The ‘Birch group’

Since Lorenz et al, 2009 a number of key studies in addition to new data, outlined herein, provide a strong justification for the inclusion of Beech, with respect to the four specified terms outlined in the EMA guidance.

Data presented herein confirms comparable structural homology and cross-reactivity of Beech pollen extract to 3-Tree positive rabbit sera with other Birch-grouped species. Table 4 summarises the characterised allergens from members of the ‘Birch group’, including Beech.

**Table 4:** **Protein families of Tree allergens in IUIS** [4]

**Protein family (PFAM)**	**Function**	**MW (kDa)**	**Beech**	**Oak**	**Birch**	**Alder**	**Hazel**
Hsp70 (PF00012)	Luminal-binding protein	70					Cor a 10
Cupin (PF00190)	11S seed storage globulin (legumin-like)	44					Cor a 9
							Cor a 11
Isoflavone reductase (PF05368)	Isoflavone reductase	34			Bet v 6		
4 EF-hand domain (PF13499)	Polcalcin-like protein	24			Bet v 3		
PR-10	Pathogenesis-related protein PR-10	17	Fag s 1	Que a 1	Bet v 1	Aln g 1	Cor a 1
Oleosin (PF01277)	Oleosin	16					Cor a 12
							Cor a 13
Profilin (PF00235)	Profilin	14			Bet v 2		Cor a 2
Prolamin (PF00234)	Non-specific lipid transfer protein 1	11					Cor a 8
EF-hand domain (PF00036)	Polcalcin	9			Bet v 4	Aln g 4	

Allergens in Oak and Beech pollen have only been partly characterised. Only Fag s 1 has been identified from Beech but this number is comparable to that of the currently grouped species - Oak, which has only Que a 1 further characterised. In addition to this, Beech performs comparably, and in some cases more convincingly, in other cross-reactivity studies [6,25,28]. As stated in Lorenz *et al.*, 2009 only a limited degree of information on the cross-reactivity between Beech and Birch pollen was available. However, a study performed by Egger *et al.*, 2008 characterised the allergen profile of Beech and Oak pollen using sera from patients in Northern Switzerland and Austria and concluded *“…Beech and oak pollen contain allergens that cross-react with the birch pollen allergens Bet v 1, Bet v 2 and Bet v 4 and with the berberine bridge enzyme-like allergen Phl p 4 from timothy grass pollen. Sera from Swiss and Austrian patients exhibited similar IgE reactivity profiles to birch, beech and oak pollen extracts… IgE reactivity to beech pollen is mainly due to cross-reactivity with birch pollen allergens.”* [6].

Circular dichroism as well as Fourier transformed infra-red spectroscopy were performed to analyse the secondary structure elements for Fag s 1. Both methods revealed very similar secondary structure elements of the investigated proteins (Fag s 1 and Bet v 1). Furthermore, Fag s 1 alpha content (%) was more closely aligned to Bet v 1 than Que a 1 (Oak) [25]. Based on its primary sequence, Fag s 1 has a predicted overall fold identical to Bet v 1. In order to confirm this, a homology model is presented herein using Birch Bet v 1 (PDB: 1BV1) as a template structure, confirming its comparable structural homology.

Hauser and colleagues, 2011 then went on to assess *in vitro* evidence of cross reactivity. Using sera of *Fagales* allergenic individuals from Austria, basophil mediator release assays were performed. Here, the protein concentration to obtain half maximal mediator release was calculated. Although the allergenic activity of Fag s 1 appeared to be slightly lower - no statistically significant difference was detected between the allergenic potential of Bet v 1 and Fag s 1 [25].

In relation to the criteria for grouping allergen extracts into homologous groups, allergen cross-reactivity and/or structural homology has been described providing justification for inclusion of Beech within the Birch group. Identical formulation and production processes of each of the candidate allergen extracts presented in this study were used, in accordance with the requirements from the EMA guidance [2].

In addition to new and current structural homology/cross-reactivity studies, the statistical analysis of tree allergen extracts shows that the protein content stability profile of Beech is statistically similar to the grouped species of Birch, Alder and Hazel. This could support the inclusion of Beech pollen in the existing ‘Birch group’ for the extrapolation of stability data, for example.

### The ‘group of sweet grasses’

Bermuda grass is not grouped with the sweet grasses of the Poaceae (Gramineae) family since cross-reactivity was not deemed as being ‘substantial’ enough [1]. While homology has a well-defined meaning (i.e. it is either present or absent), it is not clear and it is not elaborated at what level cross-reactivity is deemed substantial enough to meet the criteria set out by the EMA. Two allergens may have identical structures, but subtle differences in surface residues (i.e. substitution of similar residues) may result in differences in the affinity of IgE to bind to identical epitope regions. However, affinity may also be influenced by the degree of exposure and sensitisation of individuals by allergens in different parts of the world, which results in different clonal expansions of low, moderate or high affinity antibodies [14]. In addition to this, there is a conceptual issue when correlating structural homology and cross-reactivity. While it is true that without structural homology there would be no basis for cross-reactivity, minor changes to surface residues can have the propensity to significantly diminish IgE reactivity. This has been demonstrated between different isoforms of the same allergen, such as Bet v 1 [29]. Thus, this presents a conundrum when interpreting the current guideline. No well-defined quantitative criteria are, or can be, set with respect to species cross-reactivity, subsequently introducing a degree of subjective interpretation.

Cross-reactivity studies have since been explored including a comparison of allergen-specific IgE binding from different grass species by Johansen *et al.*, 2009, using patient sera from thousands of subjects from North America and Europe [14]. A larger dispersion of data points is observed for *C. Dactylon* (Bermuda grass) with a Spearman rank correlation factor of 0.43, compared to 0.89-0.97 for other grouped grass species. The observed difference in cross-reactivity could likely be explained by the difference in *affinity* of the IgE binding to identical epitopes from different species (i.e. Cyn d 1: Phl p 1). In some individuals that are exposed to a higher degree of different allergens from different grasses, clonal selection of high-affinity antibodies may dictate such a response, leading to multi-cross-reacting antibodies [14]. A comparison of allergen-specific IgE binding from different grass species was also assessed using a Magic Lite System [14]. It was stated that the inherent differences in surface residues have the potential to give rise to lower probability of cross-reacting IgE antibodies (as reflected by the lower sequence identity). While this may be true to some extent, the majority of solvent-accessible amino acid residues are identical, or at least similar, and present enough to harbour more common, highly conserved, IgE-binding epitopes within the group 1 allergens than unique ones.

In another study, grass pollens from 13 different species (12 of which represent the current grass homologous group) were extracted under identical conditions and yielded different profiles in terms of protein content, IgG, IgE reactivity [8]. These differences were attributed to the inherent variability between each of the grasses. The data also established structural homology and epitope sharing between species, consistent with criteria proposed in Lorenz *et al.*, 2009, thus providing sufficient data to propose the inclusion of Crested Dogstail within the sweet grasses group of the Pooideae family. The same could be proposed for that of Bermuda grass, based on this rationale.

The statistical analysis of grass extracts presented shows that the protein content stability profile of Bermuda grass was approximately the same for other grouped species. This could support the inclusion of Bermuda grass pollen in the existing ‘grass group’ for the extrapolation of stability data, for example. However, further characterisation of relevant allergens from Bermuda grass and comparison of cross-reactive patterns between subjects specifically in areas with high abundance of both Pooideae and Chloridoideae is currently lacking, especially since a number of cross-reactivity studies in this context appear to be contradictory.

## Conclusions

MS allows the possibility to identify individual proteins or allergens from complex mixes by mass and/or sequence, and has been extensively applied to the allergen field. New data on the homology, cross-reactivity and biological parameters of allergen sources have been considered to extend the work of Lorenz *et al.*, 2009 in the context of Tree and Grass species. The concept of homologous groups is certainly dynamic, allowing the flexibility and potential in streamlining quality parameters, such as stability profiles, due to extrapolation of exemplar data to a wider range of allergens. The work presented herein pays tribute to the concept of homologous groups, providing an assessment of the current literature and supporting data for the inclusion of Beech and Bermuda grass species in their respective homologous groups.

## Additional files

Additional file 1:
**Graphical display of negative control (Ragweed positive sera) assessing the cross-reactivity between the selected tree species.**
Additional file 2:
**Graphical display of negative control (Olive positive sera) assessing the cross-reactivity between the selected grass species.**

## Abbreviations

DPBS: Dulbecco’s phosphate-buffered saline
ELISA: Enzyme-linked immunosorbent assay
EMA: European medicines agency
ISAC: Internal sample attenuator counting
MS: Mass spectrometry
NCBI: National centre for biotechnology information
PDB: Protein data bank
RMSD: Root mean squared deviation
