# Supplementary figures and images for: Molecular, proteomic and immunological parameters of allergens provide inclusion criteria for new candidates within established grass and tree homologous groups
Source: World Allergy Organ J. 2015 Jul 16;8(1):21. doi: 10.1186/s40413-015-0069-9 (PMC4504082; doi:10.1186/s40413-015-0069-9)

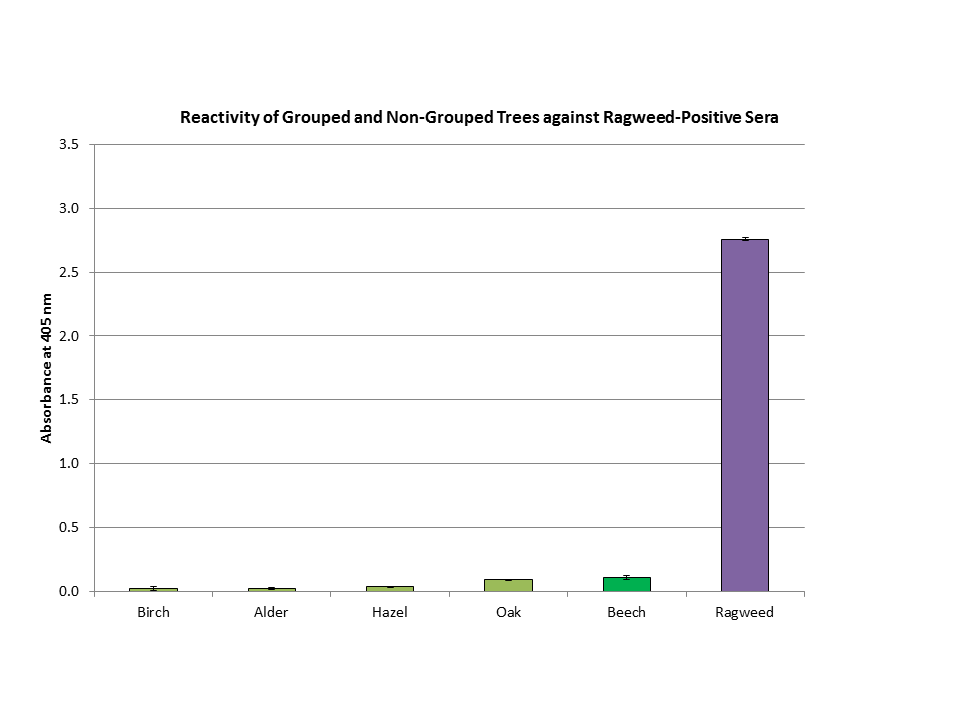

Supplement: Additional file 1: — Graphical display of negative control (Ragweed positive sera) assessing the cross-reactivity between the selected tree species. [file 40413_2015_69_MOESM1_ESM.tiff]

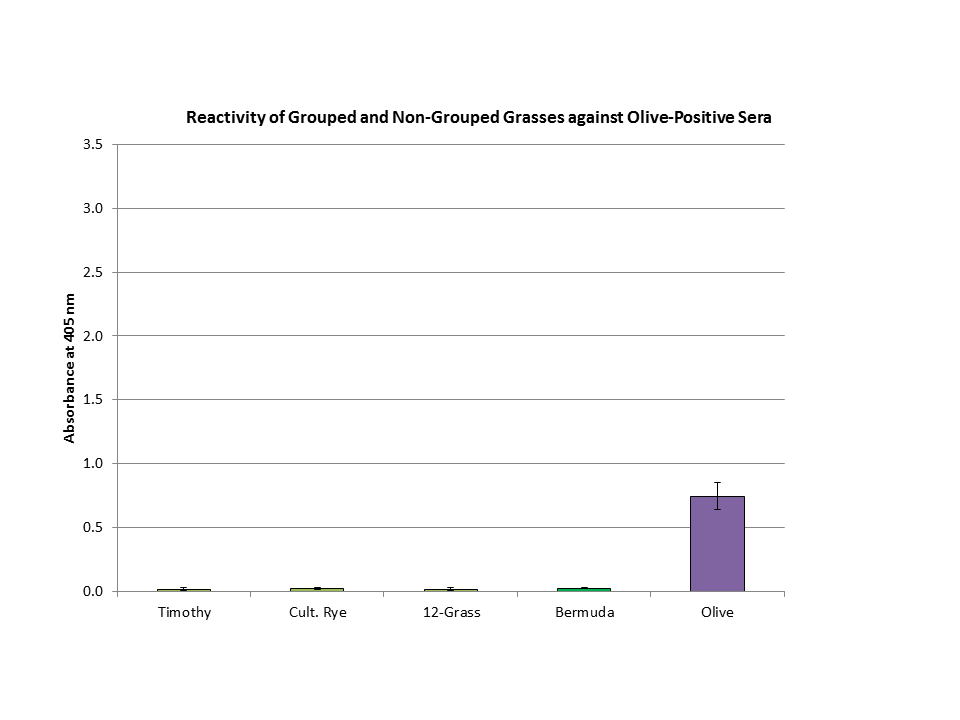

Supplement: Additional file 2: — Graphical display of negative control (Olive positive sera) assessing the cross-reactivity between the selected grass species. [file 40413_2015_69_MOESM2_ESM.tiff]
